# Mid-term follow-up results of calcaneal reconstruction for calcaneal malunion

**DOI:** 10.1186/s12891-019-2419-1

**Published:** 2019-01-29

**Authors:** Hong Seop Lee, Woo Jong Kim, Eun Seok Park, Jun Young Kim, Young Hwan Kim, Young Koo Lee

**Affiliations:** 1 Department of Foot and Ankle Surgery, Eulji Medical Center, Eulji University, 68, Hangeulbiseok-ro, Nowoungu, Seoul, 01830 Korea; 2 Department of Orthopaedic Surgery, Soonchunhyang University Hospital Cheonan, 31, Suncheonhyang 6-gil, Dongam-gu, Cheonan, Korea; 3 Department of Orthopedic Surgery, College of Medicine, Soonchunhyang University Bucheon Hospital, 1174 Jung-1-dong, Wonmi-gu, Bucheon-si, Gyunggi-do 420-767 Republic of Korea

**Keywords:** Calcaneus fracture, Calcaneus reconstruction, Mid-term follow-up, Hindfoot

## Abstract

**Background:**

We hypothesized that calcaneal reconstruction can relieve chronic pain due to calcaneal malunion. We report the mid-term follow-up results of calcaneal reconstruction for calcaneal malunion.

**Methods:**

We reviewed the records of 10 male patients (10 ft) who underwent calcaneal reconstruction for calcaneal malunion between January 2009 and July 2014 at the mid-term follow-up. Talocalcaneal height and angle, calcaneal pitch, calcaneal width, Böhler angle, Stephens classification, and Zwipp classification were evaluated by three orthopedic doctors at each visit (pre-reconstruction, post-reconstruction, and at the last follow-up).

**Results:**

The mean follow-up period was 67.1 months (range, 48–101 months). The sites of pain before reconstruction were lateral aspect (4 patients), plantar aspect (3 patients), diffuse pain (2 patients), and anterior aspect (1 patient). There was a significant difference in talocalcaneal height, talocalcaneal angle, calcaneal pitch, calcaneal width, and Böhler angle before and after reconstruction (*p* < 0.05). There was no significant difference between reconstruction and the last follow-up. Radiological measurement agreement was calculated to be moderate to strong (intraclass correlation coefficient: 0.659–0.988). Mean American Orthopedic Foot & Ankle Society Ankle and Hindfoot score improved from 66.50 ± 9.37 pre-reconstruction to 80.30 ± 8.52 at the last follow-up (*p* < 0.05). The mean visual analog scale score improved from 8.60 ± 1.43 before reconstruction to 3.40 ± 0.84 at the last follow-up (p < 0.05). Most patients were satisfied with the outcome postoperatively.

**Conclusions:**

Our results showed substantial improvement in the clinical and radiological outcomes after calcaneal reconstruction of calcaneal malunion. This outcome was maintained until the mid-term follow-up. Therefore, calcaneal reconstruction may be a good option for the treatment of chronic pain caused by the malunion of a calcaneal fracture without severe subtalar arthritis. Further prospective studies are needed to test this theory.

**Level of Evidence**: Level IV, Retrospective Case Series.

## Background

Calcaneal fractures are a common fracture in the hindfoot caused by a high energy trauma, such as a fall or motorcycle accident [1]. Ninety percent of calcaneal fractures occur in workers in their 20s to 40s [1]. Calcaneal fractures can be complicated by subtalar arthritis and malunion, leading to persistent postoperative pain and low patient satisfaction [2, 3]. Decreased Bohler’s angle is known to be a poor prognostic factor [4, 5]. Malunion is related to the primary fracture patterns. Pain associated with malunion can be variable in origin, such as peroneal tendon or lateral malleolus impingement due to widening of the calcaneus, anterior tibio-talar impingement due to a loss of height leading to a decrease in the talar inclination angle, and varus or valgus alignment causing pain during ambulation. Traditionally, corrective fusion was the mainstay treatment for malunion, and many studies have reported favorable results [6, 7]. Alternatively, subtalar joint fusion is a salvage procedure, which leads to a decrease in the range of motion and function, and it may lead to periarticular joint arthritis.

We previously reported favorable results during a short-term follow-up after calcaneal reconstructions for correction of the height and width of the malunited calcaneus [8]. In the present study, we hypothesize that calcaneal reconstructions will relieve pain for patients who have no severe subtalar arthritis but have chronic pain due to calcaneal malunion. The purpose of this study was to report the mid-term follow-up of the clinical and radiological results of calcaneal reconstruction of calcaneal malunion.

## Methods

### Patients

The inclusion criteria were as follows: diagnosis of post-calcaneus fracture malunion; previous operative history for a calcaneal fracture; calcaneal reconstruction surgery for calcaneal malunion; postoperative follow-up of a minimum of 4 years; and symptoms of postoperative pain. The exclusion criteria were patients who had severe subtalar arthritis or those who underwent subtalar arthrodesis or a simple ostectomy.

**Fig. 1:**
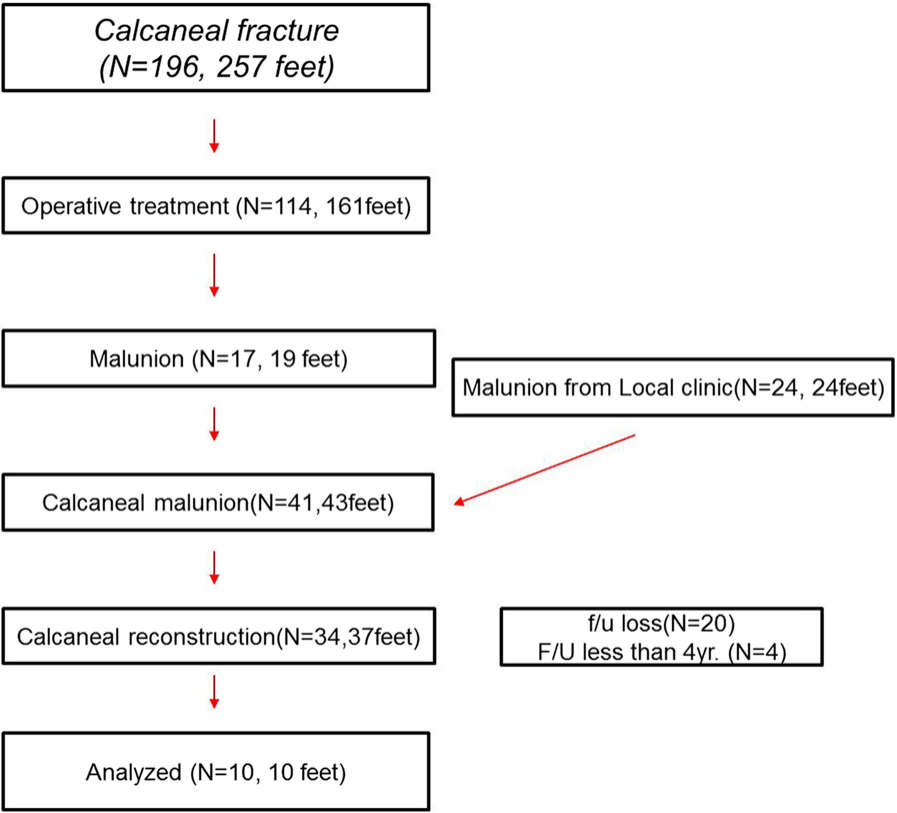
This figure shows the number of patients included in this study

Between January 2009 to July 2014, 34 patients (37 ft) underwent calcaneal reconstruction for the dysfunction and pain caused by malunion. We reviewed 10 patients (10 ft) postoperatively at the mid-term follow-up. Patient enrollment is described in Fig. 1. This study was approved by the institutional review board of our hospital. We obtained informed consent from all patients.

We evaluated the patients, including the site of pain and range of ankle motion. Radiological examinations of the foot in the weight-bearing anteroposterior and lateral views as well as a hindfoot alignment view were performed. Talocalcaneal height and angle, calcaneal pitch, calcaneal width, and the Böhler angle were measured three times by three different orthopedic doctors at each of the visits (pre-reconstruction, post-reconstruction, and final follow-up). In addition, three different orthopedic doctors classified patients according to the Stephens classification and Zwipp classification at each of these visits [9, 10]. Lateral wall bumpectomy was performed in patients with lateral impingement or without significant loss in calcaneal height; the remaining patients underwent calcaneal reconstruction involving calcaneal osteotomy followed by repositioning and fixation of the calcaneal tuberosity fragment. Postoperatively, a short leg splint was applied for 2 weeks followed by another 2 weeks with a short leg cast. After full weight-bearing was possible, postoperative shoes were used for 2 months. Subtalar fusion was performed in patients with persistent subtalar pain, no improvement in symptoms, or a limited range of motion. The American Orthopedic Foot & Ankle Society (AOFAS) Ankle and Hindfoot score and visual analog pain scale (VAS) were obtained postoperatively and at the final follow-up. The subjective satisfaction of each patient was assessed with a survey.

### Operative technique

**Fig. 2:**
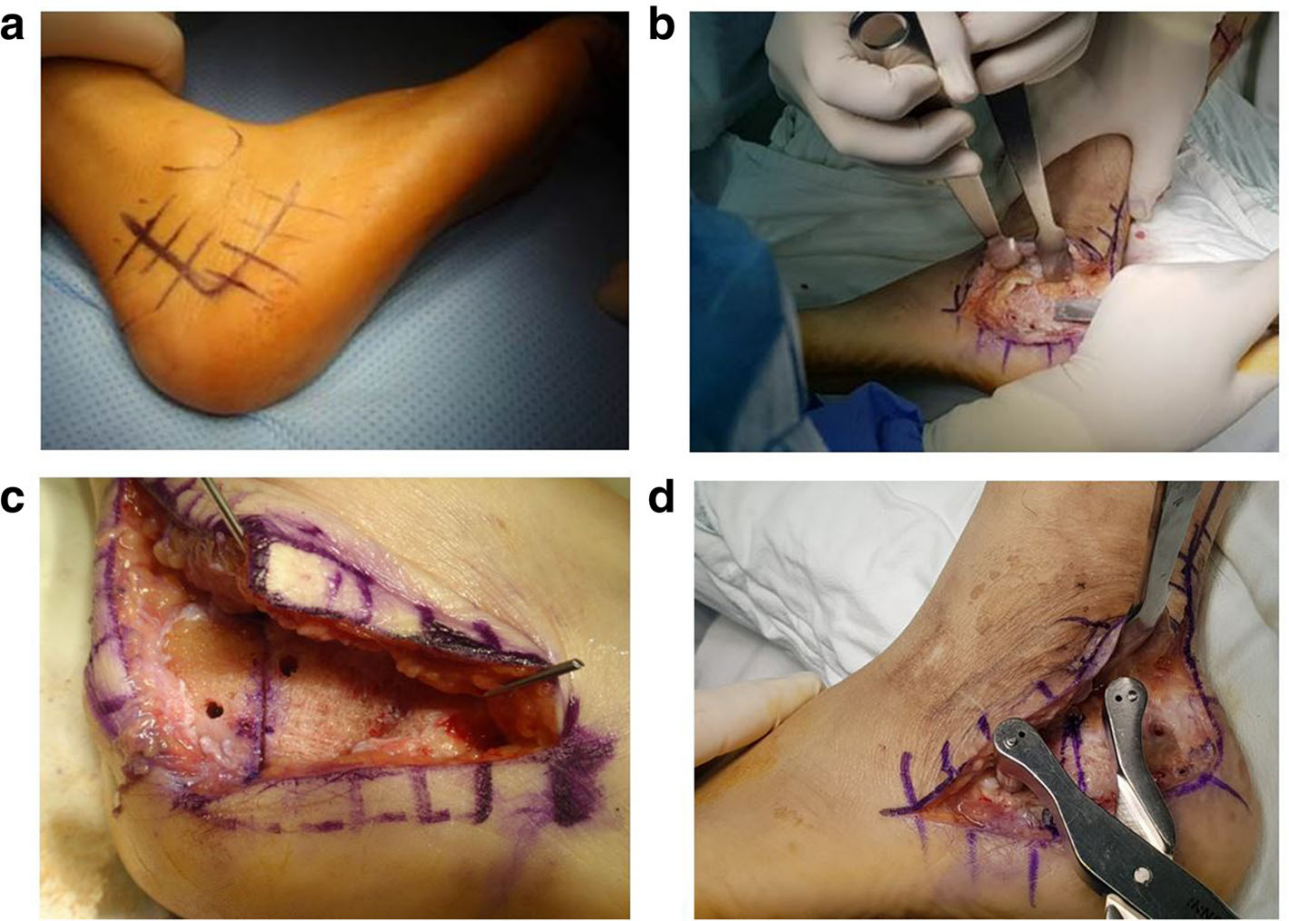
**a**. A standard extensile lateral approach is marked on the calcaneus. **b**. A lateral bumpectomy is carried out. **c**. A downward oblique osteotomy is done on the calcaneal lateral wall. **d**. Two temporary pin fixations are fixed on the calcaneal lateral wall and the tuberosity fragment is slid downward using a compressor

**Fig. 3:**
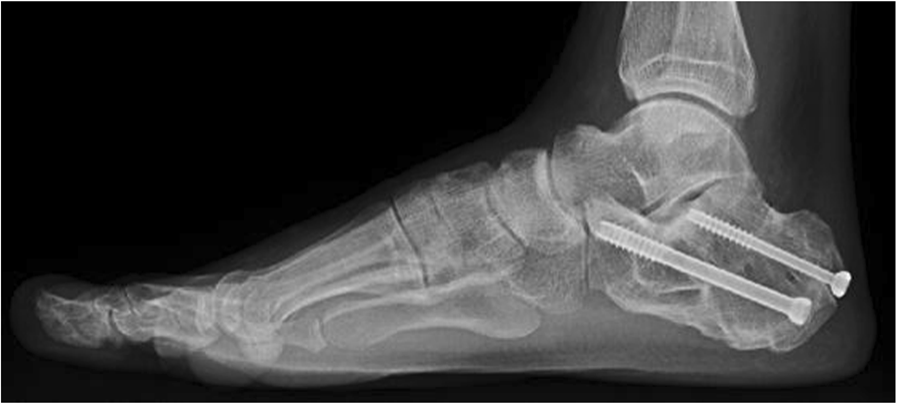
The osteotomy site is fixed with two 6.5 mm cannulated screws

Patients were placed in the lateral decubitus position on a beanbag, with the foot to be operated on facing upward. We applied a thigh tourniquet, which was inflated to 300 mmHg. We used the standard extensile lateral approach to the calcaneus. An incision was made on the vertical limb just anterior to the Achilles tendon, allowing the sural nerve to be protected within the full-thickness flap. We took care not to injure the sural nerve at the distal end of the incision. Three 1.6-mm Kirschner wires were inserted into the lateral malleoli, talar neck, and the cuboid to protect the peroneal tendons and the full-thickness flap. We performed a lateral wall bumpectomy if impingement syndrome was present in the subfibular area. A downward oblique osteotomy was started from the calcaneal lateral wall and proceeded to the medial calcaneal wall. Two temporary pin fixations on the calcaneal lateral wall and the tuberosity fragment were slid downward using a compressor (Fig. 2). Finally, we fixed the osteotomy site in the correct position with two 6.5 mm cannulated screws and staples (Fig. 3).

### Statistical analysis

The Statistical Package for the Social Sciences version 20.0 (IBM Corp., Armonk, NY, USA) was used for the statistical analysis. The McNemar test, paired *t* test, and intraclass correlation coefficient were used. The sample size was calculated using the PS program [11]. Considering previous studies, we assumed that the minimum clinically significant difference in the mean AOFAS Ankle and Hindfoot score would be 8, ranging 6–10 standard deviations between the baseline and follow-up [8]. We calculated a sample size of 9 to detect the minimum clinically significant difference with power of 80% and an alpha error of 5%.

## Results

The study included 10 male patients with a mean age of 46.3 years (range, 31–6 years). The mean interval between the first operation and the reconstruction surgery was 16.6 months (range, 7–59 months). The mean follow-up period was 67.1 months (range, 48–101 months) after calcaneal reconstruction. The sites of pain before the reconstructive operation were the lateral aspect (4 patients), plantar aspect (3 patients), diffuse pain (2 patients), and anterior aspect (1 patient).

The results of the radiological measurements and classifications by three different orthopedic doctors are summarized in Table 1 (A, B, and C). There was a significant difference in talocalcaneal height, talocalcaneal angle, calcaneal pitch, calcaneal width, and Böhler angle before and after reconstruction (*p* < 0.05). No significant differences were observed between reconstruction and the final follow-up.

**Table 1:** Results of radiologic measurements and classifications by three different orthopedic doctors

	Pre-reconstruction period	Post-reconstruction period		Post-reconstruction period	last follow up	
	Number of patients	Number of patients	*p*-value	Number of patients	Number of patients	p-value
Stephens classification			0.072			0.564
Type I	1	4		4	3	
Type II	2	5		5	6	
Type III	7	1		1	1	
Zwipp classification			0.199			0.513
Type 1	0	4		4	3	
Type 2	2	2		2	4	
Type 3	7	4		4	3	
Type 4	1	0		0	0	
Type 5	0	0		0	0	
	Mean ± SD	Mean ± SD	p-value	Mean ± SD	Mean ± SD	p-value
Talocalcaneal Height(mm)	73.17 ± 4.42	80.83 ± 3.66	< 0.05	80.83 ± 3.66	80.00 ± 0.2	0.352
Talocalcaneal Angle (°)	29.69 ± 4.99	37.55 ± 5.36	< 0.05	37.55 ± 5.36	38.40 ± 5.28	0.724
Calcaneal Pitch(°)	17.24 ± 5.07	23.74 ± 4.26	< 0.05	23.74 ± 4.26	24.40 ± 4.17	0.663
Calcaneal Width(mm)	43.46 ± 4.89	41.60 ± 5.45	< 0.05	41.60 ± 5.45	41.90 ± 5.92	0.603
Bohler Angle(°)	6.9 ± 10.09	17.79 ± 10.43	< 0.05	17.90 ± 10.44	17.40 ± 10.62	0.296
	Pre-reconstruction period	Post-reconstruction period		Post-reconstruction period	last follow up	
	Number of patients	Number of patients	p-value	Number of patients	Number of patients	p-value
Stephens classification			0.112			0.564
Type I	1	4		4	3	
Type II	3	5		5	6	
Type III	6	1		1	1	
Zwipp classification			0.199			0.513
Type 1	0	4		4	3	
Type 2	2	2		2	4	
Type 3	7	4		4	3	
Type 4	1	0		0	0	
Type 5	0	0		0	0	
	Mean ± SD	Mean ± SD	p-value	Mean ± SD	Mean ± SD	p-value
Talocalcaneal Height(mm)	71.49 ± 5.95	78.44 ± 2.74	< 0.05	78.44 ± 2.74	78.06 ± 2.59	0.509
Talocalcaneal Angle (°)	29.38 ± 5.43	30.54 ± 5.01	< 0.05	30.54 ± 5.01	30.37 ± 7.06	0.889
Calcaneal Pitch(°)	17.10 ± 6.14	23.60 ± 5.47	< 0.05	23.60 ± 5.47	22.66 ± 5.45	0.069
Calcaneal Width(mm)	42.94 ± 5.93	40.92 ± 5.50	< 0.05	40.92 ± 5.50	41.19 ± 6.03	0.526
Bohler Angle(°)	6.20 ± 10.51	19.11 ± 11.83	< 0.05	19.11 ± 11.83	18.80 ± 11.50	0.484
	Pre-reconstruction period	Post-reconstruction period		Post-reconstruction period	last follow up	
	Number of patients	Number of patients	p-value	Number of patients	Number of patients	p-value
Stephens classification			0.072			0.999
Type I	1	4		4	4	
Type II	3	5		5	5	
Type III	6	1		1	1	
Zwipp classification			0.363			0.317
Type 1	1	4		4	4	
Type 2	3	2		2	1	
Type 3	5	4		4	5	
Type 4	1	0		0	0	
Type 5	0	0		0	0	
	Mean ± SD	Mean ± SD	p-value	Mean ± SD	Mean ± SD	p-value
Talocalcaneal Height(mm)	74.20 ± 6.05	78.20 ± 6.07	< 0.05	78.20 ± 6.07	77.00 ± 4.40	0.479
Talocalcaneal Angle (°)	33.50 ± 7.44	35.90 ± 7.03	< 0.05	35.90 ± 7.03	38.00 ± 4.62	0.305
Calcaneal Pitch(°)	19.10 ± 5.26	24.70 ± 5.74	< 0.05	24.70 ± 5.74	24.30 ± 5.21	0.494
Calcaneal Width(mm)	42.88 ± 5.96	40.27 ± 6.25	< 0.05	40.27 ± 6.25	40.71 ± 6.28	0.303
Bohler Angle(°)	5.80 ± 10.42	18.30 ± 12.26	< 0.05	18.30 ± 12.26	18.81 ± 12.37	0.315

A. Results of first orthopedic doctor

The radiological measurement agreement among the three observers was calculated using the intraclass correlation coefficient, which was found to be moderate to strong (0.659–0.990) (Table 2).

**Table 2:** Radiologic agreements using Intraclass Correlation Coefficient

	Value of ICC (95%CI)		
	Pre reconstruction	Post reconstruction	Last follow up
Talocalcaneal Height(mm)	0.953 (0.862–0.987)	0.697 (0.114–0.918)	0.659 (0.001–0.908)
Talocalcaneal Angle(°)	0.858 (0.585–0.962)	0.550(−0.318–0.878)	0.923 (0.774–0.979)
Calcaneal Pitch(°)	0.926 (0.785–0.980)	0.886 (0.67–0.969)	0.859 (0.586–0.962)
Calcaneal Width(mm)	0.969 (0.908–0.992)	0.912 (0.756–0.969)	0.982 (0.946–0.995)
Bohler Angle(°)	0.981 (0.944–0.995)	0.990 (0.969–0.997)	0.988 (0.966–0.997)

*ICC* Intraclass Correlation Coefficient, *CI* Confidence Interval

At the final follow-up, 3 patients were very satisfied, 5 patients were satisfied, and 2 patients were not satisfied. In addition, the mean AOFAS Ankle and Hindfoot score improved from 66.50 ± 9.37 pre-reconstruction to 80.30 ± 8.52 at the final follow-up (*p* < 0.05) (Table 3). The mean VAS score improved from 8.60 ± 1.43 pre-reconstruction to 3.40 ± 0.84 at the final follow-up (p < 0.05). Although the pain did not completely resolve, most of the patients were satisfied postoperatively.

**Table 3:** AOFAS Ankle and Hindfoot score change between pre-reconstruction and last follow-up

AOFAS score	Pre-reconstruction	Last follow-up	p-value
Pain	23.5 ± 3.57	30.7 ± 4.10	< 0.05
Function	35.1 ± 5.81	40.7 ± 6.78	< 0.05
Alignment	6.4 ± 2.10	8.9 ± 2.50	< 0.05
Overall	66.50 ± 9.37	80.30 ± 8.52	< 0.05

*AOFAS* American Orthopedic Foot & Ankle Society

In the postoperative period, superficial wound infection occurred in 4 of the 37 ft (10.8%). Infections resolved with oral antibiotics and simple dressings and did not develop into further complications.

## Discussion

The most important finding of this study was that there was a statistically significant improvement in the radiographical and clinical outcome of calcaneal reconstruction for calcaneal malunion at the mid-term follow-up. Satisfactory short-term follow-up results were also previously reported in 10 patients with an average follow-up period of 14 months [12]. In our former study on 24 patients with an average follow-up period of 11.2 months, favorable results in the radiographic parameters and patient subjective satisfaction were observed [8]. To the best of our knowledge, this is the first study to report on the mid-term follow-up results for calcaneal reconstruction of calcaneal malunion.

The principle goal of our calcaneal reconstruction was improvement of the talocalcaneal relationship through restoration of calcaneal height. Restoration of calcaneal height relieves the anterior tibiotalar impingement as the horizontal talus is changed to vertical. Decreased Achilles tendon lever-arm due to calcaneal height loss is also improved through restoration of calcaneal height.

The optimal treatment of calcaneal fractures is still controversial among orthopedic surgeons [13]. A recent prospective, randomized, controlled multicenter study reported that in short term follow-up, operative treatment for displaced intra-articular calcaneal fracture was not superior to non-surgical treatment, however, in midterm follow-up, there were several favorable results for surgical repair [14]. An economic evaluation study comparing operative and nonoperative management of intra-articular displaced calcaneal fractures reported that operative management was more economical [15]. Anatomic reduction and firm fixation results in favorable outcomes in terms of subjective patient’s satisfaction, early rehabilitation, painful subtalar arthritis, and subtalar fusion rate [16–18]. Another prospective, randomized, controlled multicenter study reported that non-operative treatment for displaced intra-articular calcaneal fractures had equivalent functional results to those after operative treatment [19]. However, both operative and non-operative treatment can result in symptomatic malunion with severe functional disability that may impact the patient’s quality of life [5, 20].

To the best our knowledge, there is no report on effective nonoperative treatment for calcaneal malunion. For all patients, nonoperative treatment for calcaneal malunion with customized insole use for at least 6 months failed. The morphological changes of the calcaneal malunion do not improve with nonoperative treatment including orthotics and insoles.

Several studies have associated the pathological cause of the symptoms in calcaneal malunion with the pattern of the calcaneal fracture [15, 16]. They explained that a primary fracture coursing superolateral to inferomedial or anterolateral to posteromedial caused lateral and proximal displacement of the tuberosity fragment, leading to a loss of height, widening of the heel, and lateral impingement.

Recently, Savva et al. reported the results of in situ arthrodesis with lateral wall ostectomy for complication of calcaneal fractures [21]. They recommended in situ subtalar arthrodesis with lateral wall ostectomy for subtalar arthritis following calcaneal malunion, regardless of the degree of calcaneal height loss. Their results suggested that anterior tibiotalar impingement is not a significant problem after subtalar fusion. Clare et al. reported the intermediate- to long-term results of their operative protocol for calcaneal malunion [22]. They proposed an operative protocol based on Stephens classification. Since restoration of calcaneal height loss is difficult, they emphasized the importance of acute surgery for displaced intra-articular calcaneal fracture. Yu et al. reported the clinical and radiologic outcomes of reconstructive osteotomy and bone graft for calcaneal malunions [23]. They restored the displaced posterior facet through calcaneal osteotomy and an iliac bone graft filler.

Similarly, in 1993, Romash first introduced the concept of calcaneal reconstruction based on reversing the deformity associated with the later complications [12]. Reconstructive osteotomy permits the repositioning of the tuberosity, which narrows the heel, resolves impingement, and restores heel height. Clare et al. reported a lateral closing wedge osteotomy to correct a severe varus deformity and medializing calcaneal osteotomy by rotating the tuberosity for the severe valgus deformity [22].

For the evaluation of calcaneal malunion, Böhler’s angle with a the lateral view, the talocalcaneal angle, and the height of the calcaneus are most commonly used [4, 24].] Since malunion leads to a decreased talocalcaneal angle and talar inclination angle, an increasing deformity of the calcaneus occurs, indicating tibiotalar impingement [12, 24]. Similarly, the results from our previous study also suggested a statistically significant increase in the talocalcaneal angle after calcaneal reconstruction [8].

In calcaneal malunion, etiologies can vary depending on the site of pain [1, 3]. The primary cause of lateral pain is subtalar arthrosis. Symptomatic subtalar arthrosis is characterized by the aggravation of pain with palpation of the sinus tarsi. Peroneal tendon pathology, calcaneocuboid arthrosis, hardware irritation, and sural nerve impingement can also be a source of lateral pain. Anterior pain is caused primarily by anterior talar neck impingement on the distal tibia, resulting from the loss of calcaneal height. Plantar pain is caused by plantar exostosis or heel pad injury. Medial pain may be the result of tarsal tunnel syndrome or a flexor hallucis longus tendon problem. Nerve-related problems or complex regional pain syndrome can induce poorly localized pain. In our study, the site of pain was in the lateral aspect (4 patients), plantar aspect (3 patients), diffuse pain (2 patients), and anterior aspect (1 patient). Patients with calcaneal malunion complain of various areas of pain. Restoration of calcaneal height through calcaneal reconstruction could improve anterior and plantar pain. Lateral wall bumpectomy could improve lateral pain.

There are several limitations to this study. Although we calculated a sample size on the basis of our previous study, there is a bias due to small sample size. A larger group of patients and a longer follow-up period are needed. Furthermore, our study did not involve a control group. It may be interesting to compare the clinical and radiologic changes following our reconstruction technique versus the changes seen following subtalar arthrodesis. As our study is limited to a retrospective review, further prospective studies are needed. Our technique is associated with the risk of development of subtalar arthritis in the long term, which could require subtalar arthrodesis in the future. Long-term follow-up is needed to evaluate the occurrence of subtalar arthritis in the future.

## Conclusions

Our results showed substantial improvement in the clinical and radiological outcomes after calcaneal reconstruction of calcaneal malunion. This outcome was maintained until the mid-term follow-up. Therefore, calcaneal reconstruction may be a good option for the treatment of chronic pain caused by the malunion of a calcaneal fracture without severe subtalar arthritis. Further prospective studies are needed to test this theory.

## Abbreviations

AOFAS: American Orthopedic Foot & Ankle Society
VAS: Visual Analogue Scale
